# Differential prognostic value of high-sensitivity troponin T based on renal function status: insights from 14,208 ACS patients undergoing PCI

**DOI:** 10.3389/fcvm.2025.1689234

**Published:** 2025-12-19

**Authors:** Kun Na, Xueqing Yang, Miaohan Qiu, Xi Zhang, Yi Li, Yaling Han

**Affiliations:** 1 State Key Laboratory of Frigid Zone Cardiovascular Disease, Department of Cardiology, Cardiovascular Research Institute, General Hospital of Northern Theater Command, Shenyang, China; 2 Department of Cardiology, Second Affiliated Hospital of Harbin Medical University, Harbin, Heilongjiang, China

**Keywords:** acute coronary syndrome, high-sensitivity troponin T, long-term prognosis, percutaneous coronary intervention, renal function

## Abstract

**Background and objectives:**

High-sensitivity troponin T (hsTnT) is a standard biomarker for myocardial injury detection, but its prognostic value may differ by renal function status. This study evaluated how renal function modifies the prognostic significance of hsTnT in ACS patients undergoing PCI.

**Methods:**

This study examined 14,208 acute coronary syndrome (ACS) patients who underwent percutaneous coronary intervention (PCI), stratified by renal function [estimated glomerular filtration rate (eGFR) < 60 vs. ≥60 mL/min/1.73 m^2^] and peak hsTnT levels [<5× vs. ≥5× upper reference limit (URL)]. Primary outcomes included one-year all-cause mortality and one-year incidence of ischemic events following the index PCI procedure.

**Results:**

In patients with impaired renal function, elevated hsTnT was associated with significantly increased mortality [12.84% vs. 4.29%; adjusted hazard ratio [HR] 3.63, 95% confidence interval [CI] 2.04–6.49, *P* < 0.0001] and ischemic events (10.66% vs. 4.88%; adjusted HR: 2.72, 95% CI: 1.51–4.88, *P* = 0.0008). In patients with preserved renal function, the mortality association was attenuated (1.42% vs. 0.98%; adjusted HR: 1.40, 95% CI: 0.85–2.29, *P* = 0.1864), although ischemic events remained significantly increased (2.93% vs. 1.54%; adjusted HR: 2.06, 95% CI: 1.41–2.97, *P* = 0.0002). Restricted cubic spline analysis revealed a significant non-linear relationship between hsTnT levels and mortality specifically in impaired renal function cohort (P for non-linearity = 0.0004), whereas a predominantly linear association was observed in patients with preserved renal function. A significant interaction was observed between renal function and hsTnT for mortality prediction (P for interaction = 0.0089).

**Conclusions:**

These findings indicate that renal function substantially modifies the prognostic significance of hsTnT among ACS patients post-PCI. Integrating renal function and peak hsTnT into risk assessment may help identify high-risk subgroups requiring intensified follow-up and management.

## Introduction

Acute coronary syndrome (ACS) remains a leading cause of morbidity and mortality worldwide despite continuous advances in diagnosis and management (1–3). High-sensitivity troponin assays, including high-sensitivity cardiac troponin T (hsTnT) and I (hsTnI), are widely used biomarkers for the detection of myocardial injury, with current guidelines recommending the use of high-sensitivity cardiac troponin, without specifying I or T, for the diagnosis and risk stratification of myocardial infarction (4). The adoption of high-sensitivity assays has enabled earlier and more accurate detection of myocardial injury, transforming hsTnT from a binary diagnostic marker into a multidimensional quantitative tool for risk stratification in ACS.

However, interpreting hsTnT levels in clinical practice is complex, particularly in patients with impaired renal function (5–8). Patients with chronic kidney disease (CKD) frequently exhibit chronically elevated troponin concentrations even in the absence of acute myocardial injury. This elevation is multifactorial, resulting not only from reduced renal clearance but also from chronic inflammation induced by uraemic toxins, subclinical myocardial injury, left ventricular hypertrophy, oxidative stress, and accelerated cardiovascular remodeling within the cardiorenal syndrome (9–13). This physiological overlap complicates the distinction between chronic elevation and acute myocardial injury, contributing to potential risk misclassification.

Current risk assessment models may inadequately account for the potential interaction between renal function and troponin levels (14, 15). While elevated troponin consistently predicts poor outcomes in patients with preserved renal function, its prognostic significance in the setting of renal impairment remains incompletely characterized. Most prognostic studies either exclude patients with significant renal dysfunction or fail to analyze them as a separate cohort. The relationship between hsTnT levels and clinical outcomes may not be uniform across the spectrum of renal function, potentially following non-linear patterns that current risk models fail to capture. This study therefore examines the differential prognostic implications of hsTnT levels in 14,208 ACS patients undergoing PCI, with specific focus on how renal function modifies this relationship, to develop more sophisticated risk stratification approaches for this complex patient population.

## Methods

### Study design and patients

This study was a *post-hoc* analysis of a single-center, all-comer, prospective, real-world PCI registry in the General Hospital of Northern Theater Command. From March 2016 to March 2019, consecutive ACS patients who underwent PCI were enrolled. Inclusion criteria were ACS patients aged 18 years or older who received PCI with at least one stent implanted. Patients were excluded if they underwent diagnostic angiography only, were referred for coronary artery bypass grafting (CABG) without stent placement during the index procedure, or if data necessary for calculating hsTnT or eGFR were unavailable. The study was approved by the hospital's Research Ethics Committee (K2018-35). The study complied with the provisions of the Declaration of Helsinki. Data collection was conducted using a standard web-based platform (CV-NET system of Crealife Technology, Beijing, China).

### Assessment of renal function and high-sensitivity cardiac troponin T

Baseline clinical characteristics and serum biochemistry results were collected using the electronic patient record. Serum creatinine at presentation was used to calculate the eGFR using the Modification of Diet in Renal Disease study equation: eGFR (mL/min/1.73 m^2^) = 175 × (serum creatinine)^−1.154^ (mg/dL) × age^−0.203^ × 0.742 (if female) (16). Based on this, patients were classified as having normal (eGFR ≥60 mL/min/1.73 m^2^) or impaired renal function (eGFR <60 mL/min/1.73 m^2^) (17).

Blood samples for hs-cTnT were collected at admission and at 6–12 h post-PCI. The peak value from serial measurements was used for outcome analysis, while admission levels served as baseline reference. Hs-cTnT was quantified by electrochemiluminescence immunoassay using the Elecsys high-sensitivity Troponin T assay (Roche Diagnostics) on a Cobas e 411 analyzer. The lower detection limit was ≤0.003 ng/mL, with a 99th percentile upper reference limit of 0.1 ng/mL.

### Outcomes and definitions

The primary endpoint was all-cause death at 12 months. Secondary endpoints included ischemic events at 12 months, deﬁned as a composite of cardiac death, myocardial infarction (MI), or stroke and each component of ischemic events. Cardiovascular death was defined according to the Academic Research Consortium-2 (ARC-2) consensus criteria (18), encompassing death caused by acute myocardial infarction, sudden cardiac death including unwitnessed death, heart failure, stroke, cardiovascular procedures, cardiovascular hemorrhage, and other cardiovascular causes. All deaths were adjudicated by a clinical events committee based on hospital records, death certificates. Deaths were classified as cardiovascular unless a definite non-cardiovascular cause was established; deaths of undetermined cause were classified as cardiovascular. MI was deﬁned according to the Third Universal Deﬁnition of Myocardial Infarction Guidelines (19). Stroke was deﬁned as a loss of neurologic function induced by an ischemic episode that lasted at least 24 h or resulted in mortality, as determined by clinicians or imaging investigations. Unstable angina (UA) was diagnosed in patients presenting with ischemic symptoms, no ST-segment elevation on ECG, and hs-cTnT levels below the 99th percentile upper reference limit. Non-ST-segment elevation myocardial infarction (NSTEMI) was defined as elevated hs-cTnT above the 99th percentile URL without persistent ST-segment elevation. ST-segment elevation myocardial infarction (STEMI) was defined as elevated hs-cTnT above the 99th percentile URL with ST-segment elevation ≥0.1 mV in ≥2 contiguous leads or new left bundle branch block. ACS classification was recorded in the electronic medical records and CV-NET registry system at the time of presentation (20). Left ventricular ejection fraction (LVEF) was assessed by transthoracic echocardiography during the index hospitalization, typically within 24–48 h of admission, or within 24 h post-procedure for patients undergoing primary PCI for STEMI. All patients were followed-up by telephone or email at 1, 6, and 12 months.

### Statistical analysis

Continuous variables were assessed for normality using the Shapiro–Wilk test (*n* < 5,000) or Kolmogorov–Smirnov test (*n* ≥ 5,000) (Supplementary Table S1 and Supplementary Figures S1–S7). Variables with normal distribution are presented as mean ± SD; non-normally distributed variables are presented as median (Q1–Q3). Categorical variables are displayed as counts and percentages. Baseline characteristics were compared between eGFR groups using chi-squared tests for categorical variables and Student's *t*-test or Mann–Whitney *U*-test for continuous variables, as appropriate. These comparisons served a descriptive purpose to characterize group differences rather than for confirmatory hypothesis testing, therefore, correction for multiple comparisons was not applied. The proportion of missing data for each variable is presented in Supplementary Table S2. Time-to-event data with estimated event rates measured with the Kaplan–Meier method were compared using the log-rank test. The proportional hazards assumption was tested using Schoenfeld residuals. A 30-day landmark analysis was performed as sensitivity analysis. Multivariable Cox regression models were adjusted for age, sex, hypertension, previous myocardial infarction, previous PCI, previous stroke, smoking status, type of ACS, anemia, arterial access, coronary arteries treated, and number of stents. The non-linear association between hsTnT concentration and 12-month outcomes was evaluated using restricted cubic spline (RCS) regression within the Cox proportional hazards framework, with 4 knots placed at the 5th, 35th, 65th, and 95th percentiles. Unless otherwise specified, a 2-sided *P* value <0.05 was considered to indicate statistical significance. Statistical analysis was performed using SAS software version 9.3 (SAS Institute, Cary, NC).

## Results

### Overview of the study population

**Figure 1:**
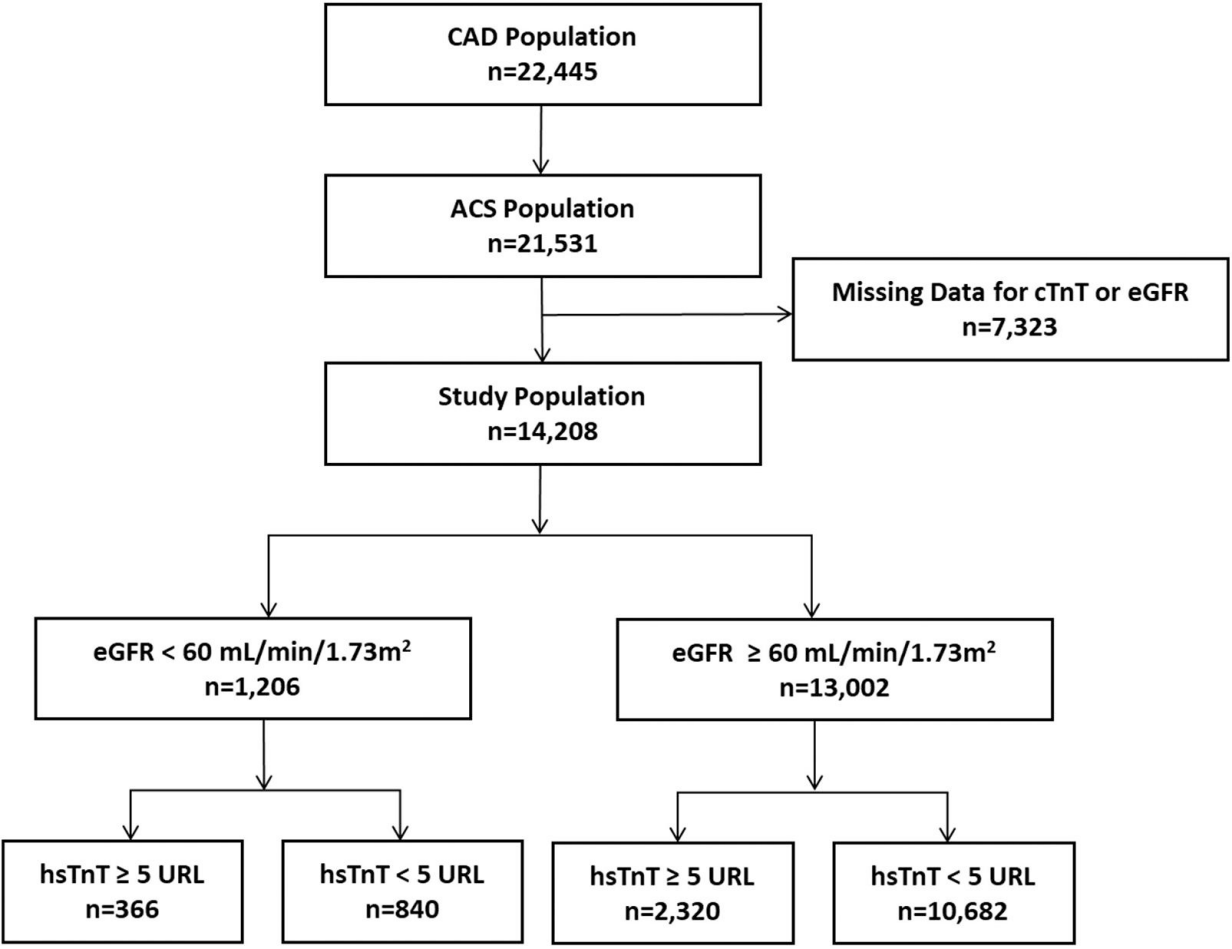
Study population flow chart. CAD, coronary artery disease; ACS, acute coronary syndrome; hsTnT, high-sensitivity troponin-T.

This investigation encompassed 14,208 ACS patients undergoing PCI, all with documented serum creatinine measurements and post-procedural high-sensitivity troponin T (hsTnT) assessments. Baseline characteristics of included and excluded patients are presented in Supplementary Table S3. Based on renal function parameters, patients were stratified into two cohorts: impaired renal function (eGFR <60 mL/min/1.73 m^2^, *n* = 1,206) and preserved renal function (eGFR ≥60 mL/min/1.73 m^2^, *n* = 13,002). Further stratification by hsTnT elevation revealed that among patients with impaired renal function, 366 subjects (30.3%) exhibited hsTnT elevations exceeding five times the 99th percentile upper reference limit (URL), while 840 (69.7%) remained below this threshold. In the preserved renal function cohort, 2,320 patients (17.8%) demonstrated hsTnT elevation ≥5 URL, with 10,682 subjects below this cutoff. The patient selection algorithm is illustrated in Figure 1.

### Study population characteristics

The eGFR was significantly higher in the preserved renal function group compared to the impaired renal function group (96.39 ± 20.11 vs. 46.03 ± 12.02 mL/min/1.73 m^2^; *P* < 0.001). Patients with impaired renal function were characterized by advanced age (68.12 ± 9.98 vs. 60.36 ± 10.15 years; *P* < 0.001) and higher female prevalence (38.14% vs. 25.65%; *P* < 0.0001). This cohort also demonstrated significantly higher rates of cardiovascular risk factors, including diabetes mellitus and hypertension, as well as greater prevalence of prior PCI, myocardial infarction, and cerebrovascular events (all *P* < 0.01). Regarding angiographic and procedural characteristics, patients with renal impairment exhibited more frequent left main coronary artery involvement (7.88% vs. 5.98%; *P* = 0.0088) and required longer stent lengths (47.17 ± 26.02 vs. 44.95 ± 26.04 mm; *P* = 0.006). Post-discharge pharmacotherapy patterns revealed higher clopidogrel utilization in the renal impairment group (76.63% vs. 67.04%; *P* < 0.0001), whereas statin (90.22% vs. 93.25%; *P* < 0.0001) and aspirin (94.20% vs. 98.21%; *P* < 0.0001) prescription rates were higher in patients with preserved renal function. Notably, post-procedural peak hsTnT values were significantly elevated in the renal impairment cohort [0.12 (0.03–0.74) vs. 0.04 (0.02–0.23) ng/L; *P* < 0.001] (Table 1). Peak hsTnT values also differed significantly across ACS subtypes: median (IQR) 0.02 (0.01–0.05) ng/L for UA, 0.14 (0.04–0.46) ng/L for NSTEMI, and 0.84 (0.15–2.43) ng/L for STEMI (*P* < 0.0001) (Supplementary Table S4). Comprehensive patient characteristics, discharge medication profiles, procedural parameters and clinical outcomes stratified by hsTnT elevation are presented in Supplementary Tables S5, S6.

**Table 1:** Baseline clinical characteristics, procedural characteristics, and medication after discharge in all patients and stratified by renal function.

Variable	Total cohort (*N* = 14,208)	eGFR < 60 (*N* = 1,206)	eGFR ≥ 60 (*N* = 13,002)	*P* value
Age, years	61.02 ± 10.36	68.12 ± 9.98	60.36 ± 10.15	<0.0001
Male	10,413 (73.29%)	746 (61.86%)	9,667 (74.35%)	<0.0001
Medical history				
Hypertension	8,839 (62.32%)	965 (80.08%)	7,874 (60.67%)	<0.0001
Diabetes	4,376 (30.89%)	511 (42.55%)	3,865 (29.81%)	<0.0001
Previous MI	2,674 (18.89%)	329 (27.46%)	2,345 (18.10%)	<0.0001
Previous PCI	3,695 (26.05%)	358 (29.73%)	3,337 (25.71%)	0.0024
Previous stroke	2,103 (14.84%)	274 (22.78%)	1,829 (14.11%)	<0.0001
Smoking				<.0001
Never	6,063 (42.84%)	627 (52.16%)	5,436 (41.97%)	
Active	6,025 (42.57%)	381 (31.70%)	5,644 (43.58%)	
Former	2,066 (14.60%)	194 (16.14%)	1,872 (14.45%)	
Anemia*	2,255 (15.87%)	555 (46.02%)	1,700 (13.07%)	<0.0001
eGFR, mL/min per 1.73 m^2^	92.18 (77.67–106.91)	49.22 (39.97–55.36)	94.51 (82.14–108.56)	<0.0001
LVEF, %	58.44 ± 8.60	54.02 ± 10.14	58.85 ± 8.32	<0.0001
hsTnT at peak, ng/L	0.05 (0.02–0.26)	0.12 (0.03–0.74)	0.04 (0.02–0.23)	<0.0001
PCI presentation				
Type of ACS				<0.0001
UA	8,555 (60.21%)	589 (48.84%)	7,966 (61.27%)	
NSTEMI	2,477 (17.43%)	262 (21.72%)	2,215 (17.04%)	
STEMI	3,176 (22.35%)	355 (29.44%)	2,821 (21.70%)	
Procedural information				
Transradial access	12,998 (91.48%)	1,020 (84.58%)	11,978 (92.12%)	<0.0001
Coronary arteries treated				
Left main artery	873 (6.14%)	95 (7.88%)	778 (5.98%)	0.0088
Left anterior descending artery	7,765 (54.65%)	630 (52.24%)	7,135 (54.88%)	0.0784
Left circumflex artery	3,521 (24.78%)	273 (22.64%)	3,248 (24.98%)	0.0713
Right coronary artery	5,251 (36.96%)	482 (39.97%)	4,769 (36.68%)	0.0236
Number of stents	1.00 (1.00–2.00)	1.00 (1.00–2.00)	1.00 (1.00–2.00)	0.7372
Total length of stents, mm	38.00 (24.00–60.00)	40.50 (26.50–62.00)	38.00 (24.00–59.00)	0.001
Average stent diameters, mm	3.04 ± 0.75	3.01 ± 1.02	3.05 ± 0.72	0.2734
Discharge prescription				
Aspirin	13,905 (97.87%)	1,136 (94.20%)	12,769 (98.21%)	<0.0001
P2Y_12_ inhibitors				<0.0001
Clopidogrel	9,592 (67.85%)	915 (76.63%)	8,677 (67.04%)	
Ticagrelor	4,545 (32.15%)	279 (23.37%)	4,266 (32.96%)	
Statins	13,213 (93.00%)	1,088 (90.22%)	12,125 (93.25%)	<0.0001
ACEI/ARB	9,421 (66.31%)	769 (63.76%)	8,652 (66.54%)	0.0508
*β*blockers	9,799 (68.97%)	856 (70.98%)	8,943 (68.78%)	0.1147

Values are mean ± SD or No. (%). MI, myocardial infarction; PCI, percutaneous coronary intervention; hsTnT, high-sensitivity troponin-T; ACS, acute coronary syndrome; UA, unstable angina; STEMI, ST-segment-elevation myocardial infarction; NSTEMI, non-ST-segment-elevation myocardial infarction; LVEF, left ventricular ejection fraction; ACEI/ARB, angiotensin-converting enzyme inhibitor/angiotensin II receptor blocker.

* Anemia was deﬁned as hemoglobin <13 g/dL for men or <12 g/dL for women.

### Clinical outcomes according to eGFR

Compared to the patients with normal renal function low-risk group (eGFR ≥ 60 mL/min/1.73 m^2^), all-cause mortality (6.88% vs. 1.06%, *P* < 0.0001) within one year after PCI was significantly higher in patients with abnormal renal function (eGFR < 60 mL/min/1.73 m^2^). Significant differences were also found in the incidence of the risk for ischemic events (6.63% vs. 1.78%, *P* < 0.0001), which was mainly for the risk of cardiac death (5.31% vs. 0.74%, *P* < 0.0001). There were no significant differences in the incidence of MI (0.75% vs. 0.56%, *P* = 0.4176) and stroke (0.91% vs. 0.56%, *P* = 0.1286) between the groups (Table 2).

**Table 2:** Clinical outcomes at 1 year stratified by renal function.

Outcome	Total cohort (*N* = 14,208)	eGFR < 60 (*N* = 1,206)	eGFR ≥ 60 (*N* = 13,002)	*P* value
All-cause death	221 (1.56%)	83 (6.88%)	138 (1.06%)	<0.0001
Ischemic events	312 (2.20%)	80 (6.63%)	232 (1.78%)	<0.0001
Cardiac death	160 (1.13%)	64 (5.31%)	96 (0.74%)	<0.0001
MI	82 (0.58%)	9 (0.75%)	73 (0.56%)	0.4176
Stroke	84 (0.59%)	11 (0.91%)	73 (0.56%)	0.1286

Values are No. (%). MI, myocardial infarction. Ischemic events were defined as a composite of cardiac death, all MI, and/or stroke.

Within the impaired renal function group (eGFR <60 mL/min/1.73 m^2^), the majority of patients (63.3%) had eGFR 45–59 mL/min/1.73 m^2^, while only 28 patients (2.3%) had eGFR <15 mL/min/1.73 m^2^. One-year all-cause mortality rates increased with declining eGFR: 4.59% for eGFR 45–59, 9.18% for eGFR 30–44, 14.55% for eGFR 15–29, and 14.29% for eGFR <15 mL/min/1.73 m^2^. Similar patterns were observed for ischemic events (Supplementary Table S7).

### Clinical outcomes according to hsTnT and eGFR

**Figure 2:**
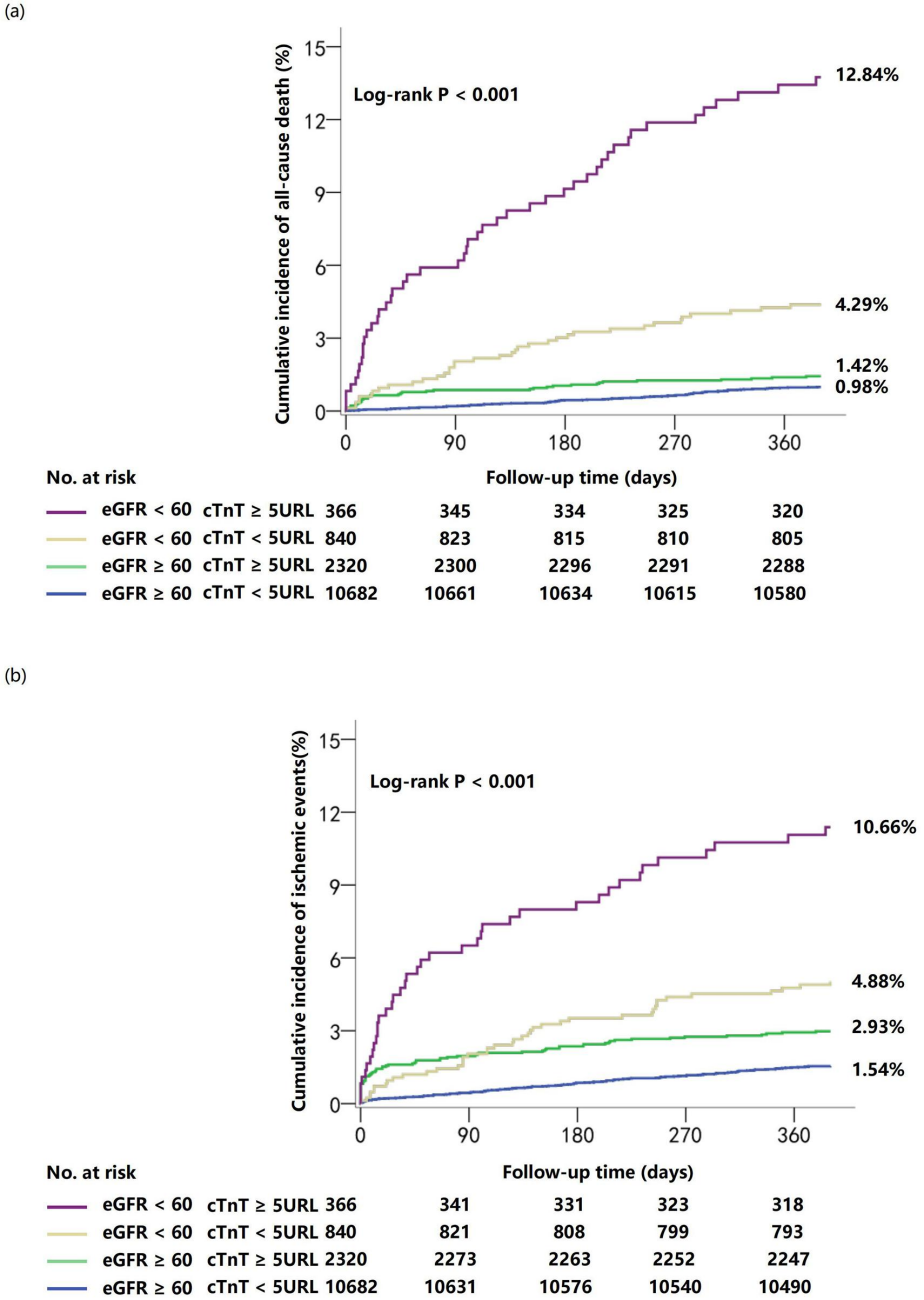
Cumulative incidence of clinical outcomes at 1 year stratified by troponin T and renal function. Curves depict cumulative incidence of all-cause death **(a)** and ischemic events **(b)**.

The analysis revealed a significant non-linear relationship between high-sensitivity troponin T levels and clinical outcomes, with distinct patterns emerging across different renal function categories. One-year cumulative incidence of all-cause mortality was highest in patients with eGFR <60 mL/min/1.73 m^2^ and hsTnT ≥5 URL (12.84%), followed by eGFR <60 mL/min/1.73 m^2^ and hsTnT <5 URL (4.29%), eGFR ≥60 mL/min/1.73 m^2^ and hsTnT ≥5 URL (1.42%), and eGFR ≥60 mL/min/1.73 m^2^ and hsTnT <5 URL (0.98%; log-rank *P* < 0.001). Similar patterns were observed for 1-year ischemic events (10.66%, 4.88%, 2.93%, and 1.54%, respectively; log-rank *P* < 0.001) (Figure 2).

**Figure 3:**
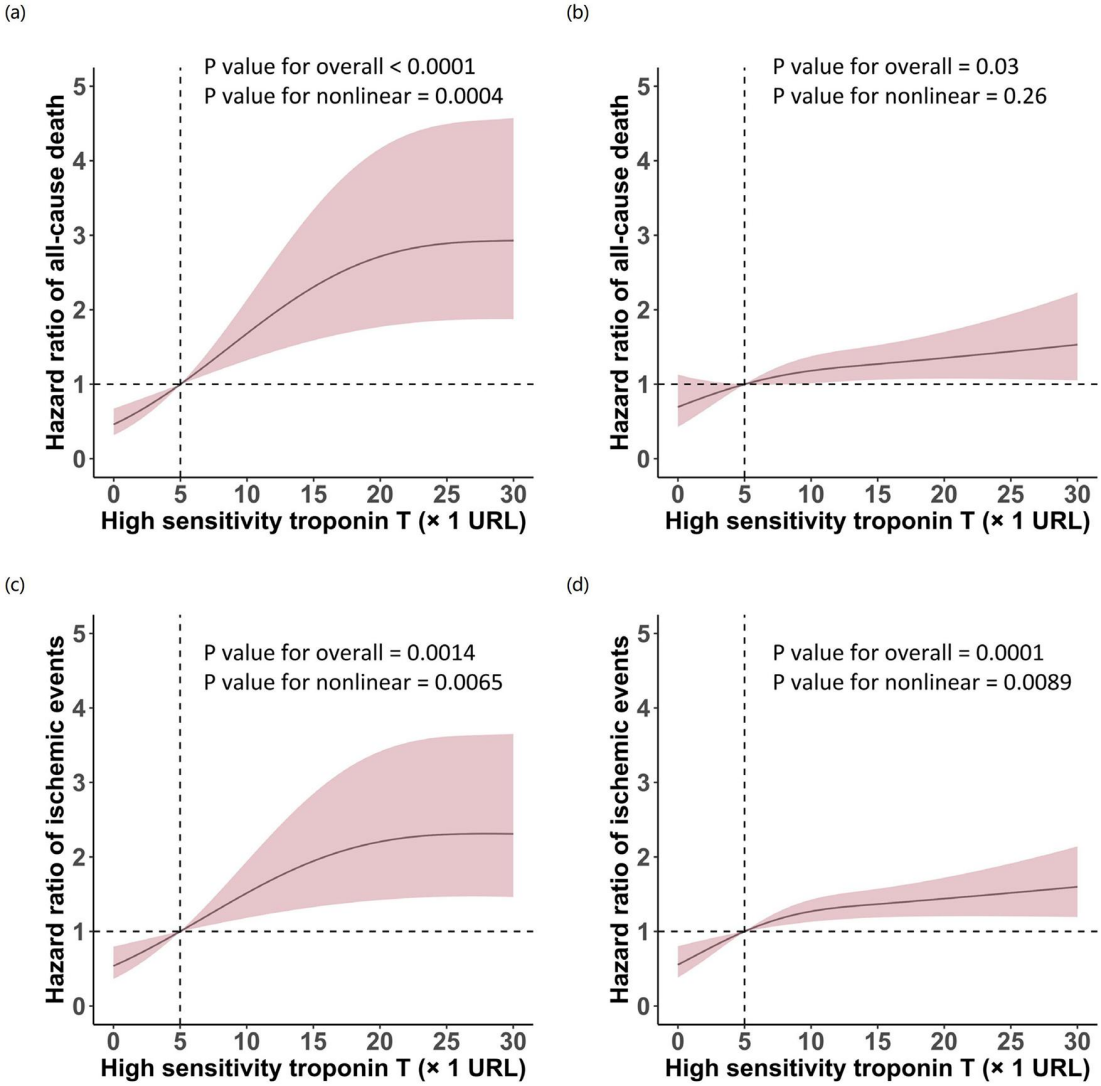
Restricted cubic spline analysis of the association between high-sensitivity troponin T levels and clinical outcomes stratified by renal function. Curves depict hazard ratios for all-cause death **(a,b)** and ischemic events **(c,d)** in patients with eGFR <60 mL/min/1.73 m^2^ **(a,c)** and eGFR ≥60 mL/min/1.73 m^2^ **(b,d)**. Solid lines represent hazard ratios; shaded areas represent 95% confidence intervals. The vertical dashed line indicates hsTnT = 5 URL.

RCS regression analysis quantified this non-linear relationship, demonstrating a significant non-linear association between hsTnT levels and all-cause mortality in patients with impaired renal function (P for overall <0.0001, P for non-linearity = 0.0004). In contrast, patients with normal renal function exhibited a predominantly linear relationship (P for overall = 0.0307, P for non-linearity = 0.2647). This differential pattern indicates that mortality risk accelerates disproportionately with increasing hsTnT levels in the context of renal impairment. Notably, the relationship between hsTnT and ischemic events maintained non-linearity regardless of renal function status, though with distinct curve morphologies (impaired renal function: P for overall = 0.0014, P for non-linearity = 0.0065; preserved renal function: P for overall = 0.0001, P for non-linearity = 0.0089) (Figure 3).

Multivariable Cox regression models confirmed a significant interaction between renal function and hsTnT levels for 1-year mortality. In patients with impaired renal function, elevated troponin conferred a 3.63-fold increase in adjusted 1-year mortality risk (aHR: 3.63; 95% CI: 2.04–6.49, *P* < 0.001), while in those with preserved renal function, this association was attenuated and did not reach statistical significance (aHR: 1.40; 95% CI: 0.85–2.29, *P* = 0.1864; P for interaction = 0.0089). This interaction effect was not observed for composite ischemic events (Pinteraction = 0.4041), but reemerged for cardiac death specifically, where elevated hsTnT demonstrated significant prognostic value only in patients with renal impairment (Table 3). These non-linear relationships and their differential patterns across renal function strata provide important insights for clinical risk assessment strategies.

**Table 3:** Adjusted associations between peak cardiac troponin T concentration and clinical outcomes at 1 year stratified by renal function.

Outcomes	hsTnT		Hazard ratio (95% CI)	*P* value	Adjusted hazard ratio (95% CI)	*P* value	*P* value for interaction
	≥5 URL	<5 URL					
All-cause death							0.0089
eGFR < 60	12.84% (47/366)	4.29% (36/840)	3.14 (2.04–4.85)	<0.0001	3.63 (2.04–6.49)	<0.0001	
eGFR ≥ 60	1.42% (33/2,320)	0.98% (105/10,682)	1.46 (0.98–2.15)	0.0599	1.40 (0.85–2.29)	0.1864	
Ischemic events							0.4041
eGFR < 60	10.66% (39/366)	4.88% (41/840)	2.29 (1.48–3.56)	0.0002	2.72 (1.51–4.88)	0.0008	
eGFR ≥ 60	2.93% (68/2,320)	1.54% (164/10,682)	1.93 (1.46–2.57)	<0.0001	2.06 (1.41–2.97)	0.0002	
Cardiac death							0.1031
eGFR < 60	9.84% (36/366)	3.33% (28/840)	3.09 (1.88–5.06)	<0.0001	3.26 (1.69–6.29)	0.0004	
eGFR ≥ 60	1.16% (27/2,320)	0.65% (69/10,682)	1.81 (1.16–2.83)	0.0089	1.55 (0.88–2.75)	0.1323	
MI							0.0821
eGFR < 60	0.55% (2/366)	0.83% (7/840)	0.66 (0.14–3.17)	0.6022	1.04 (0.15–7.35)	0.9680	
eGFR ≥ 60	1.34% (31/2,320)	0.39% (42/10,682)	3.42 (2.15–5.44)	<0.0001	3.03 (1.60–5.73)	0.0007	
Stroke							0.3475
eGFR < 60	1.09% (4/366)	0.83% (7/840)	1.32 (0.39–4.50)	0.6592	1.76 (0.34–9.02)	0.4999	
eGFR ≥ 60	0.43% (10/2,320)	0.59% (63/10,682)	0.73 (0.38–1.43)	0.3576	1.13 (0.49–2.60)	0.7798	

Values are No. (%). hsTnT, high-sensitivity troponin-T; MI, myocardial infarction. Ischemic events were defined as a composite of cardiac death, all MI, and/or stroke. Adjusted for age, sex, hypertension, previous myocardial infarction, previous PCI, previous stroke, smoking status, type of ACS, anemia, arterial access, coronary arteries treated, and number of stents.

A 30-day landmark analysis was performed, excluding 45 deaths and 87 ischemic events occurring within 30 days. After landmark, the proportional hazards assumption was satisfied (all-cause death: *P* = 0.086; ischemic events: *P* = 0.19). For all-cause mortality, event rates were 0.92% (98/10,675) in the eGFR ≥60/hsTnT <5 URL group, 0.78% (18/2,305) in the eGFR ≥60/hsTnT ≥5 URL group (HR = 0.85, 95% CI: 0.51–1.41, *P* = 0.528), 3.37% (28/832) in the eGFR <60/hsTnT <5 URL group (HR = 3.72, 95% CI: 2.45–5.67, *P* < 0.001), and 9.12% (32/351) in the eGFR <60/hsTnT ≥5 URL group (HR = 10.43, 95% CI: 7.00–15.54, *P* < 0.001). For ischemic events, event rates were 1.30% (139/10,657), 1.36% (31/2,283; HR = 1.04, 95% CI: 0.71–1.54, *P* = 0.834), 3.85% (32/831; HR = 3.01, 95% CI: 2.05–4.42, *P* < 0.001), and 6.57% (23/350; HR = 5.29, 95% CI: 3.40–8.22, *P* < 0.001), respectively (Supplementary Table S8).

## Discussion

In this retrospective study, we evaluated the prognostic value of hsTnT in ACS patients undergoing PCI, stratified by renal function. The main findings were as follows: (1) patients with impaired renal function (eGFR < 60 mL/min/1.73 m^2^) exhibited significantly higher one-year all-cause mortality and incidence of ischemic events compared to those with normal renal function (eGFR ≥ 60 mL/min/1.73 m^2^); (2) elevated hsTnT levels (≥5 URL) were more prevalent in patients with impaired renal function and were strongly associated with adverse outcomes; (3) RCS regression analysis indicated a nonlinear relationship between hsTnT levels and one-year all-cause mortality in patients with impaired renal function, while the relationship was linear in those with normal renal function. These results highlight the importance of considering renal function when interpreting hsTnT levels in ACS patients post-PCI.

Several studies have elucidated the prognostic significance of hsTnT across diverse clinical scenarios. Pareek et al. (21) demonstrated that serial hsTnT measurements during hospitalization for suspected ACS could predict both short-term and long-term mortality outcomes. However, the specific implications of hsTnT levels in ACS patients undergoing PCI, particularly when stratified by renal function, remain partially explored. These results corroborate previous findings that hsTnT levels are elevated in CKD patients and associated with increased cardiovascular events and mortality. While prior studies examined ambulatory CKD populations without acute coronary events or longitudinal cTnT in advanced CKD, the present study extends these observations to ACS patients undergoing PCI, demonstrating that the prognostic value of post-procedural hsTnT is significantly modified by renal function status (5–7). Moreover, our study reveals the differential impact of elevated hsTnT on mortality and ischemic events between patients with normal and impaired renal function. While elevated hsTnT levels were associated with increased one-year all-cause mortality and ischemic events in both groups, a significant non-linear association was observed in patients with impaired renal function. This underscores the importance of personalized risk assessment in managing ACS patients after PCI, highlighting the pronounced impact of renal health on prognosis.

The enhanced prognostic significance of elevated hsTnT in patients with impaired renal function likely reflects the capacity of troponin to integrate both chronic and acute components of cardiovascular injury in this population. Chronic kidney disease is characterized by a constellation of pathophysiological alterations, including left ventricular hypertrophy, diffuse myocardial fibrosis, microvascular ischemia, volume and pressure overload, systemic inflammation, and reduced renal clearance of troponin degradation products, all of which contribute to persistent troponin release and elevated baseline hsTnT concentrations (5–7, 12–15). Within this context, an elevated peak hsTnT level during ACS-related PCI admission may represent the cumulative burden of chronic cardiorenal stress superimposed upon acute ischemic insult, thereby identifying patients with diffuse structural heart disease and advanced vascular pathology who face particularly elevated mortality risk. Conversely, in patients with preserved renal function, baseline hsTnT concentrations are characteristically low, and elevations predominantly reflect the acute ischemic burden of the index event and periprocedural myocardial injury. After adjustment for clinical risk factors and procedural characteristics, the residual prognostic gradient across peak hsTnT levels becomes more modest in this population, consistent with the largely linear association and attenuated adjusted hazard ratios observed in our analysis. This mechanistic framework provides a coherent explanation for why the relationship between hsTnT and mortality exhibits a steeper, non-linear pattern in the presence of renal impairment, whereas it remains flatter and less discriminative when renal function is preserved.

The renal function-dependent prognostic heterogeneity of hsTnT observed in our study supports a more individualized approach to risk stratification, whereby high-risk patients with concurrent renal impairment and elevated troponin may be prioritized for intensified surveillance and early intervention. Our findings also underscore the importance of optimizing post-PCI pharmacotherapy in patients with impaired renal function. The higher clopidogrel utilization coupled with lower statin and aspirin prescription rates observed in this population suggests potential opportunities for pharmacological optimization to mitigate the elevated risk of adverse outcomes. Notably, elevated hsTnT levels (≥5 URL) were more prevalent in patients with impaired renal function compared to those with preserved renal function, consistent with the established pathophysiological impact of renal insufficiency on cardiac biomarker kinetics. Beyond the mechanisms discussed above, recent Mendelian randomization evidence has demonstrated a causal relationship between urinary sodium-potassium ratio and myocardial infarction risk (22). Given that renal dysfunction substantially alters urinary electrolyte homeostasis, this finding provides an additional mechanistic pathway linking impaired renal function to adverse cardiovascular outcomes and may partly explain the amplified prognostic significance of elevated troponin in this population.

Several clinical implications emerge from our findings. First, current risk stratification tools such as the GRACE and TIMI scores do not incorporate renal function-specific interpretation of troponin values; our data suggest that the prognostic weight of a given hsTnT elevation differs substantially depending on baseline eGFR, and future iterations of these scores may benefit from including an interaction term or renal function-stratified coefficients for troponin. Second, rather than applying uniform hsTnT thresholds across all patients, clinicians might consider lower thresholds to identify high-risk individuals among those with eGFR <60 mL/min/1.73 m^2^, given the steeper mortality gradient observed in this subgroup. Third, patients presenting with the combination of impaired renal function and elevated hsTnT may warrant intensified post-discharge management, including earlier follow-up (within 2 weeks rather than 4–6 weeks), aggressive optimization of guideline-directed medical therapy with renal-appropriate dosing, and lower thresholds for referral to cardiac rehabilitation or advanced heart failure services. Future prospective studies should validate these findings and evaluate whether incorporation of renal function-adjusted troponin thresholds into clinical decision support tools improves risk discrimination and patient outcomes.

### Limitations

Several limitations must be acknowledged. First, the retrospective nature of the study introduces potential biases, and the single-center design may limit generalizability. Second, both serum creatinine and hs-cTnT measurements have timing-related limitations: creatinine was measured at presentation, which may be affected by acute cardiorenal syndrome and may not accurately reflect baseline renal function, although hs-cTnT was routinely measured at admission and 6–12 h post-PCI per institutional protocol, precise data on time from symptom onset or balloon inflation to blood sampling were not recorded, and individual variability in sampling timing may influence peak troponin values. Third, patients with eGFR <60 mL/min/1.73 m^2^ represent a heterogeneous group, only 2.32% had eGFR <15 mL/min/1.73 m^2^, and data on renal replacement therapy were unavailable, limiting generalizability to patients with end-stage renal disease. Fourth, patients with impaired renal function had a higher burden of cardiovascular comorbidities, and unstable angina was more prevalent in patients with preserved renal function, which may influence troponin distribution. Although multivariable Cox regression was adjusted for these factors and the significant interaction between renal function and hsTnT persisted (P for interaction = 0.0089), residual confounding from unmeasured comorbidities cannot be entirely excluded. Finally, the proportional hazards assumption showed deviation in the primary analysis due to higher early event rates in high-risk patients; landmark sensitivity analysis confirmed consistent findings (Supplementary Table S8). Future prospective studies are needed to confirm these findings and explore the underlying mechanisms driving the interaction between hsTnT levels and renal function.

## Conclusion

Our findings demonstrate that renal function fundamentally transforms the prognostic significance of elevated troponin T in ACS patients undergoing PCI. This critical interaction, where elevated troponin carries three-fold greater mortality risk in renal impairment but minimal impact with normal function, establishes the necessity for integrated risk assessment incorporating both parameters to identify patients requiring intensified surveillance and intervention.

## Data Availability

The raw data supporting the conclusions of this article will be made available by the authors upon reasonable request. Requests to access the datasets should be directed to Yaling Han (hanyaling@163.net).
