# Supplementary material for: Differential prognostic value of high-sensitivity troponin T based on renal function status: insights from 14,208 ACS patients undergoing PCI
Source: Front Cardiovasc Med. 2025 Dec 19;12:1689234. doi: 10.3389/fcvm.2025.1689234 (PMC12757341; doi:10.3389/fcvm.2025.1689234)

**Differential Prognostic Value of High-Sensitivity Troponin T Based on Renal Function Status: Insights from 14,208 ACS Patients Undergoing PCI**

Supplementary material

This appendix complements the main manuscript and provides the results from additional analyses.

**Supplementary Tables**

**Table S1.** Distribution and normality assessment of continuous variables according to eGFR category. 2

**Table S2.** Missing data for baseline variables. 3

**Table S3.** Baseline Clinical Characteristics Comparing Included and Excluded Patient. 5

**Table S4.** High-Sensitivity Troponin T Values by ACS Diagnosis. 7

**Table S5.** Baseline Clinical Characteristics, Procedural Characteristics, and Medication after Discharge in All Patients and Stratified by Peak Cardiac Troponin T Concentration. 8

**Table S6.** Clinical Outcomes at 1 Year Stratified by Peak Cardiac Troponin T Concentration. 10

**Table S7.** Distribution and Clinical Outcomes Within the Impaired Renal Function Group (eGFR <60 mL/min/1.73m²). 11

**Table S8.** Landmark Analysis at 30 Days: Associations Between Renal Function and hsTnT Stratification with Clinical Outcomes. 12

**Supplementary Figures**

**Figure S1.** Histograms and Q-Q plots for age according to eGFR categories. 13

**Figure S2.** Histograms and Q-Q plots for eGFR according to eGFR categories. 14

**Figure S3.** Histograms and Q-Q plots for LVEF according to eGFR categories. 15

**Figure S4.** Histograms and Q-Q plots for hsTnT at peak according to eGFR categories. 16

**Figure S5.** Histograms and Q-Q plots for number of stents according to eGFR categories. 17

**Figure S6.** Histograms and Q-Q plots for total length of stents according to eGFR categories. 18

**Figure S7.** Histograms and Q-Q plots for average stent diameters according to eGFR categories. 19

**Table S1. Distribution and normality assessment of continuous variables according to eGFR category.**

| **Variable** | **eGFR Group** | **N** | **Normality test** | **P value** | **Approx. normal** |
| --- | --- | --- | --- | --- | --- |
| Age, years | eGFR< 60 | 1,206 | Shapiro–Wilk | <0.001 | No |
| Age, years | eGFR≥ 60 | 13,002 | K-S | <0.001 | No |
| Age, years | Overall | 14,208 | K-S | <0.001 | No |
| eGFR, mL/min/1.73 m^2^ | eGFR< 60 | 1,206 | Shapiro–Wilk | <0.001 | No |
| eGFR, mL/min/1.73 m^2^ | eGFR≥ 60 | 13,002 | K-S | <0.001 | No |
| eGFR, mL/min/1.73 m^2^ | Overall | 14,208 | K-S | <0.001 | No |
| LVEF, % | eGFR< 60 | 1,027 | Shapiro–Wilk | <0.001 | No |
| LVEF, % | eGFR≥ 60 | 10,931 | K-S | <0.001 | No |
| LVEF, % | Overall | 11,958 | K-S | <0.001 | No |
| hsTnT at peak, ng/L | eGFR< 60 | 1,206 | Shapiro–Wilk | <0.001 | No |
| hsTnT at peak, ng/L | eGFR≥ 60 | 13,002 | K-S | <0.001 | No |
| hsTnT at peak, ng/L | Overall | 14,208 | K-S | <0.001 | No |
| Number of stents | eGFR< 60 | 1,206 | Shapiro–Wilk | <0.001 | No |
| Number of stents | eGFR≥ 60 | 13,002 | K-S | <0.001 | No |
| Number of stents | Overall | 14,208 | K-S | <0.001 | No |
| Total length of stents, mm | eGFR< 60 | 1,106 | Shapiro–Wilk | <0.001 | No |
| Total length of stents, mm | eGFR≥ 60 | 12,402 | K-S | <0.001 | No |
| Total length of stents, mm | Overall | 13,508 | K-S | <0.001 | No |
| Average stent diameters, mm | eGFR< 60 | 1,106 | Shapiro–Wilk | <0.001 | No |
| Average stent diameters, mm | eGFR≥ 60 | 12,402 | K-S | <0.001 | No |
| Average stent diameters, mm | Overall | 13,508 | K-S | <0.001 | No |

**Table S2. Missing data for baseline variables.**

| **Variable** | **Missing, n (%)** |
| --- | --- |
| Age, years | 0 (0.00%) |
| Male | 0 (0.00%) |
| Medical history |  |
| Hypertension | 24 (0.17%) |
| Diabetes | 41 (0.29%) |
| Previous MI | 52 (0.37%) |
| Previous PCI | 26 (0.18%) |
| Previous stroke | 41 (0.29%) |
| Smoking | 54 (0.38%) |
| Never |  |
| Active |  |
| Former |  |
| Type of ACS | 0 (0.00%) |
| UA |  |
| NSTEMI |  |
| STEMI |  |
| Anemia* | 0 (0.00%) |
| eGFR, mL/min per 1.73 m2 | 0 (0.00%) |
| LVEF, % | 2250 (15.84%) |
| Procedural information |  |
| Transradial access | 0 (0.00%) |
| Coronary arteries treated |  |
| Left main artery | 0 (0.00%) |
| Left anterior descending artery | 0 (0.00%) |
| Left circumflex artery | 0 (0.00%) |
| Right coronary artery | 0 (0.00%) |
| Number of stents | 0 (0.00%) |
| Total length of stents, mm | 700 (4.93%) |
| Average stent diameters, mm | 700 (4.93%) |
| **Discharge prescription** |  |
| Aspirin | 0 (0.00%) |
| P2Y_12_ inhibitors | 71 (0.50%) |
| Clopidogrel |  |
| Ticagrelor |  |
| Statins | 0 (0.00%) |
| ACEI/ARB | 0 (0.00%) |
| βblockers | 0 (0.00%) |

Values are mean±SD or No. (%). Abbreviation: MI, myocardial infarction; PCI, percutaneous coronary intervention; hsTnT, high-sensitivity troponin-T; ACS, acute coronary syndrome; UA, unstable angina; STEMI, ST-segment-elevation myocardial infarction; NSTEMI, non-ST-segment-elevation myocardial infarction; LVEF, left ventricular ejection fraction; ACEI/ARB, angiotensin-converting enzyme inhibitor/angiotensin II receptor blocker.

*Anemia was deﬁned as hemoglobin <13 g/dl for men or <12 g/dl for women.

## **Table S3. Baseline Clinical Characteristics Comparing Included and Excluded Patient.**

|  | **Included**  **(N=14,208)** | **Excluded**  **(N=7,323)** | **P value** |
| --- | --- | --- | --- |
| Age, years | 61.02±10.36 | 60.50±10.32 | <0.001 |
| Male | 10,413 (73.29%) | 5,386 (73.55%) | 0.683 |
| ***Medical history*** |  |  |  |
| Hypertension | 8,839 (62.32%) | 4,506 (61.62%) | 0.322 |
| Diabetes | 4,376 (30.89%) | 2,338 (32.02%) | 0.089 |
| Previous MI | 2,674 (18.89%) | 1,326 (18.16%) | 0.196 |
| Previous PCI | 3,695 (26.05%) | 2,024 (27.70%) | 0.010 |
| Previous stroke | 2,103 (14.84%) | 966 (13.23%) | 0.001 |
| Smoking |  |  | 0.310 |
| Never | 6,063 (42.84%) | 3,052 (41.87%) |  |
| Active | 6,025 (42.57%) | 3,180 (43.63%) |  |
| Former | 2,066 (14.60%) | 1,057 (14.50%) |  |
| Anemia* | 2,255 (15.87%) | 1,090 (17.36%) | <0.001 |
| LVEF, % | 58.44±8.60 | 57.60±8.91 | <0.001 |
| Type of ACS |  |  | <0.001 |
| UA | 8,555 (60.21%) | 4,179 (57.07%) |  |
| NSTEMI | 2,477 (17.43%) | 1,295 (17.69%) |  |
| STEMI | 3,176 (22.35%) | 1,849 (25.25%) |  |
| Transradial access | 12,998 (91.48%) | 6,794 (92.78%) | <0.001 |
| Coronary arteries treated |  |  |  |
| Left main artery | 873 (6.14%) | 300 (4.10%) | <0.001 |
| Left anterior descending artery | 7,765 (54.65%) | 3,633 (49.61%) | <0.001 |
| Left circumflex artery | 3,521 (24.78%) | 1,625 (22.19%) | <0.001 |
| Right coronary artery | 5,251 (36.96%) | 2,884 (39.38%) | <0.001 |
| Number of stents | 1.00 (1.00-2.00) | 1.00 (1.00-2.00) | <0.001 |
| Total length of stents, mm | 38.00 (24.00-60.00) | 36.00 (24.00-56.00) | <0.001 |
| Average stent diameters, mm | 3.04±0.75 | 3.04±0.71 | 0.826 |
| ***Discharge prescription*** |  |  |  |
| Aspirin | 13,905 (97.87%) | 7,112 (97.12%) | <0.001 |
| P2Y_12_ inhibitors |  |  | 0.030 |
| Clopidogrel | 9,592 (67.85%) | 4,764 (66.38%) |  |
| Ticagrelor | 4,545 (32.15%) | 2,413 (33.62%) |  |
| Statins | 13,213 (93.00%) | 6,681 (91.23%) | <0.001 |
| ACEI/ARB | 9,421 (66.31%) | 4,597 (62.77%) | <0.001 |
| β-blockers | 9,799 (68.97%) | 5,019 (68.54%) | 0.518 |

Values are mean±SD or No. (%). Abbreviation: MI, myocardial infarction; PCI, percutaneous coronary intervention; hsTnT, high-sensitivity troponin-T; ACS, acute coronary syndrome; UA, unstable angina; STEMI, ST-segment-elevation myocardial infarction; NSTEMI, non-ST-segment-elevation myocardial infarction; LVEF, left ventricular ejection fraction; ACEI/ARB, angiotensin-converting enzyme inhibitor/angiotensin II receptor blocker.

*Anemia was deﬁned as hemoglobin <13 g/dl for men or <12 g/dl for women.

**Table S4. High-Sensitivity Troponin T Values by ACS Diagnosis.**

| **ACS Type** | **N** | **hsTnT at peak, ng/L, median (IQR)** | **P value** |
| --- | --- | --- | --- |
|  |  |  | <0.0001 |
| UA | 8,555 | 0.02 (0.01–0.05) |  |
| NSTEMI | 2,477 | 0.14 (0.04–0.46) |  |
| STEMI | 3,176 | 0.84 (0.15–2.43) |  |

**Abbreviations**: ACS, acute coronary syndrome; UA, unstable angina; NSTEMI, non-ST-segment-elevation myocardial infarction; STEMI, ST-segment-elevation myocardial infarction; hsTnT, high-sensitivity troponin T; IQR, interquartile range.

**Table S5. Baseline Clinical Characteristics, Pprocedural Characteristics, and Medication after Discharg in All Patients and Stratiffed by Peak Cardiac Troponin T Concentration.**

|  | <1 × URL (N=9014) | 1-5 × URL (N=2508) | ≥5 × URL (N=2686) | P value |
| --- | --- | --- | --- | --- |
| Age, years | 60.94±9.90 | 61.14±10.55 | 61.17±11.64 | 0.2134 |
| Male | 6492 (72.02%) | 1878 (74.88%) | 2043 (76.06%) | <.0001 |
| Medical history |  |  |  |  |
| Hypertension | 5793 (64.37%) | 1570 (62.65%) | 1476 (55.10%) | <.0001 |
| Diabetes | 2874 (31.98%) | 796 (31.81%) | 706 (26.37%) | <.0001 |
| Previous MI | 1961 (21.84%) | 428 (17.11%) | 285 (10.65%) | <.0001 |
| Previous PCI | 2789 (31.00%) | 582 (23.21%) | 324 (12.09%) | <.0001 |
| Previous stroke | 1321 (14.70%) | 375 (14.98%) | 407 (15.21%) | 0.7846 |
| Smoking |  |  |  | <.0001 |
| Never | 4032 (44.92%) | 1066 (42.61%) | 965 (36.06%) |  |
| Active | 3447 (38.40%) | 1132 (45.24%) | 1446 (54.04%) |  |
| Former | 1497 (16.68%) | 304 (12.15%) | 265 (9.90%) |  |
| Type of ACS |  |  |  | <.0001 |
| UA | 7336 (81.38%) | 973 (38.80%) | 246 (9.16%) |  |
| NSTEMI | 1043 (11.57%) | 851 (33.93%) | 583 (21.71%) |  |
| STEMI | 635 (7.04%) | 684 (27.27%) | 1857 (69.14%) |  |
| Anemia* | 1158 (12.85%) | 526 (20.97%) | 571 (21.26%) | <.0001 |
| eGFR, mL/min per 1.73 m2 | 94.96±23.21 | 89.56±25.33 | 84.96±23.93 | <.0001 |
| LVEF, % | 60.38±7.74 | 57.37±8.77 | 53.10±8.66 | <.0001 |
| Procedural information |  |  |  |  |
| Transradial access | 8308 (92.17%) | 2271 (90.55%) | 2419 (90.06%) | 0.0005 |
| Coronary arteries treated |  |  |  |  |
| Left main artery | 556 (6.17%) | 215 (8.57%) | 102 (3.80%) | <.0001 |
| Left anterior descending artery | 5076 (56.31%) | 1386 (55.26%) | 1303 (48.51%) | <.0001 |
| Left circumflex artery | 2263 (25.11%) | 731 (29.15%) | 527 (19.62%) | <.0001 |
| Right coronary artery | 3233 (35.87%) | 946 (37.72%) | 1072 (39.91%) | 0.0005 |
| Number of stents | 1.63±0.87 | 1.78±0.98 | 1.35±0.79 | <.0001 |
| Total length of stents, mm | 45.19±26.15 | 50.91±28.17 | 39.31±21.91 | <.0001 |
| Average stent diameters, mm | 3.05±0.83 | 3.01±0.55 | 3.04±0.60 | 0.0504 |
| Discharge prescription |  |  |  |  |
| Aspirin | 8888 (98.60%) | 2467 (98.37%) | 2550 (94.94%) | <.0001 |
| P2Y_12_ inhibitors |  |  |  | <.0001 |
| Clopidogrel | 6272 (69.78%) | 1654 (66.43%) | 1666 (62.66%) |  |
| Ticagrelor | 2716 (30.22%) | 836 (33.57%) | 993 (37.34%) |  |
| Statins | 8472 (93.99%) | 2331 (92.94%) | 2410 (89.72%) | <.0001 |
| ACEI/ARB | 5893 (65.38%) | 1747 (69.66%) | 1781 (66.31%) | 0.0003 |
| βblockers | 6256 (69.40%) | 1786 (71.21%) | 1757 (65.41%) | <.0001 |

Values are mean±SD or No. (%). Abbreviation: MI, myocardial infarction; PCI, percutaneous coronary intervention; hsTnT, high-sensitivity troponin-T; ACS, acute coronary syndrome; UA, unstable angina; STEMI, ST-segment-elevation myocardial infarction; NSTEMI, non-ST-segment-elevation myocardial infarction; LVEF, left ventricular ejection fraction; ACEI/ARB, angiotensin-converting enzyme inhibitor/angiotensin II receptor blocker.

*Anemia was deﬁned as hemoglobin <13 g/dl for men or <12 g/dl for women.

**Table S6. Clinical Outcomes at 1 Year Stratiffed by Peak Cardiac Troponin T Concentration.**

|  | <1 × URL (N=9014) | 1-5 × URL (N=2508) | ≥5 × URL (N=2686) | P value |
| --- | --- | --- | --- | --- |
| Ischemic events | 148 (1.64%) | 57 (2.27%) | 107 (3.98%) | <0.0001 |
| Cardiac death | 63 (0.70%) | 34 (1.36%) | 63 (2.35%) | <0.0001 |
| MI | 38 (0.42%) | 11 (0.44%) | 33 (1.23%) | <0.0001 |
| Stroke | 55 (0.61%) | 15 (0.60%) | 14 (0.52%) | 0.8689 |
| All-cause death | 96 (1.07%) | 45 (1.79%) | 80 (2.98%) | <0.0001 |

Values are No. (%). Abbreviation: MI, myocardial infarction. Ischemic events were defined as a composite of cardiac death, all MI, and /or stroke.

**Table S7. Distribution and Clinical Outcomes Within the Impaired Renal Function Group (eGFR <60 mL/min/1.73m²).**

| **eGFR subgroup (mL/min/1.73m²)** | **N (%)** | **All-cause death, n**  **(%)** | **Ischemic events, n**  **(%)** |
| --- | --- | --- | --- |
| 45–59 | 763 (63.27%) | 35 (4.59%) | 36 (4.72%) |
| 30–44 | 305 (25.29%) | 28 (9.18%) | 26 (8.52%) |
| 15–29 | 110 (9.12%) | 16 (14.55%) | 14 (12.73%) |
| <15 | 28 (2.32%) | 4 (14.29%) | 4 (14.29%) |

Abbreviations: eGFR, estimated glomerular filtration rate.

**Table S8. Landmark Analysis at 30 Days: Associations Between Renal Function and hsTnT Stratification with Clinical Outcomes.**

| **Outcome** | **eGFR**  **(mL/min/1.73m²)** | **hsTnT** | **N** | **Events, n**  **(%)** | **HR (95% CI)** | **P value** |
| --- | --- | --- | --- | --- | --- | --- |
| **All-cause death** |  |  |  |  |  |  |
|  | ≥60 | <5 URL | 10,675 | 98 (0.92%) | Reference | — |
|  | ≥60 | ≥5 URL | 2,305 | 18 (0.78%) | 0.85 (0.51–1.41) | 0.528 |
|  | <60 | <5 URL | 832 | 28 (3.37%) | 3.72 (2.45–5.67) | <0.001 |
|  | <60 | ≥5 URL | 351 | 32 (9.12%) | 10.43 (7.00–15.54) | <0.001 |
| **Ischemic events** |  |  |  |  |  |  |
|  | ≥60 | <5 URL | 10,657 | 139 (1.30%) | Reference | — |
|  | ≥60 | ≥5 URL | 2,283 | 31 (1.36%) | 1.04 (0.71–1.54) | 0.834 |
|  | <60 | <5 URL | 831 | 32 (3.85%) | 3.01 (2.05–4.42) | <0.001 |
|  | <60 | ≥5 URL | 350 | 23 (6.57%) | 5.29 (3.40–8.22) | <0.001 |

**Note:** Landmark analysis excluded patients with events within 30 days (45 deaths and 87 ischemic events). Proportional hazards assumption was satisfied after landmark (all-cause death: P=0.086; ischemic events: P=0.19). **Abbreviations:** eGFR, estimated glomerular filtration rate; hsTnT, high-sensitivity troponin T; URL, upper reference limit; HR, hazard ratio; CI, confidence interval.

**Figure S1. Histograms and Q-Q plots for age according to eGFR categories.**


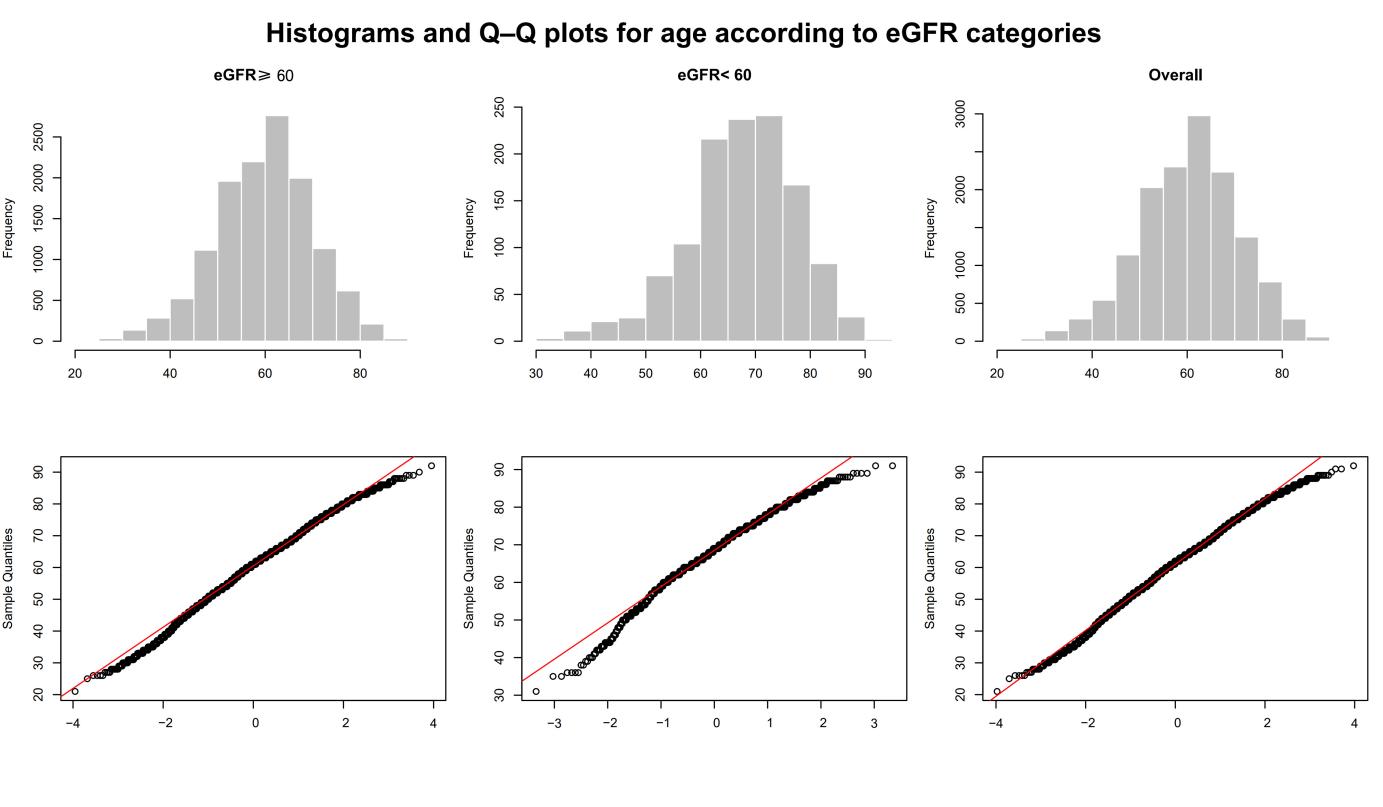


**Figure S2. Histograms and Q-Q plots for eGFR according to eGFR categories.**


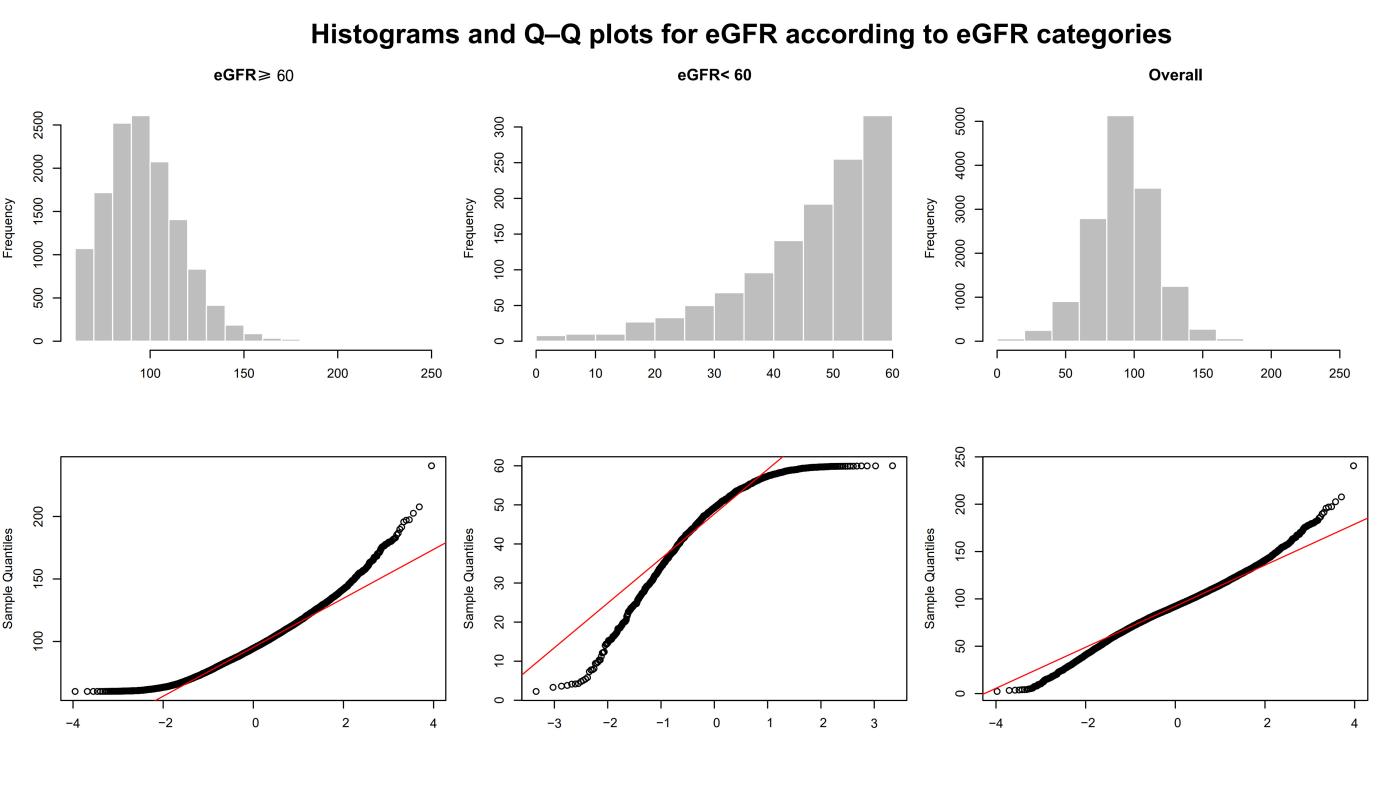


**Figure S3. Histograms and Q-Q plots for LVEF according to eGFR categories.**

##
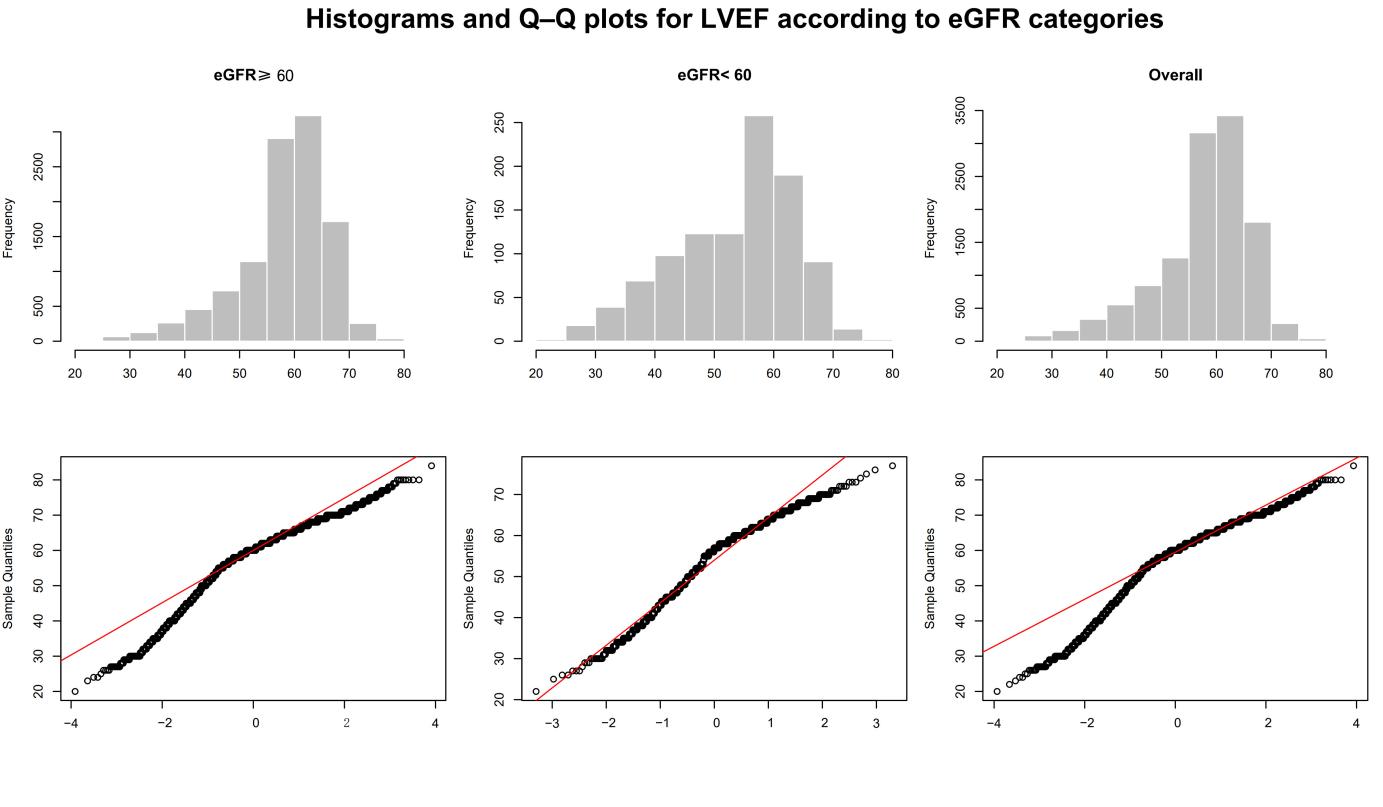


**Figure S4. Histograms and Q-Q plots for hsTnT at peak according to eGFR categories.**


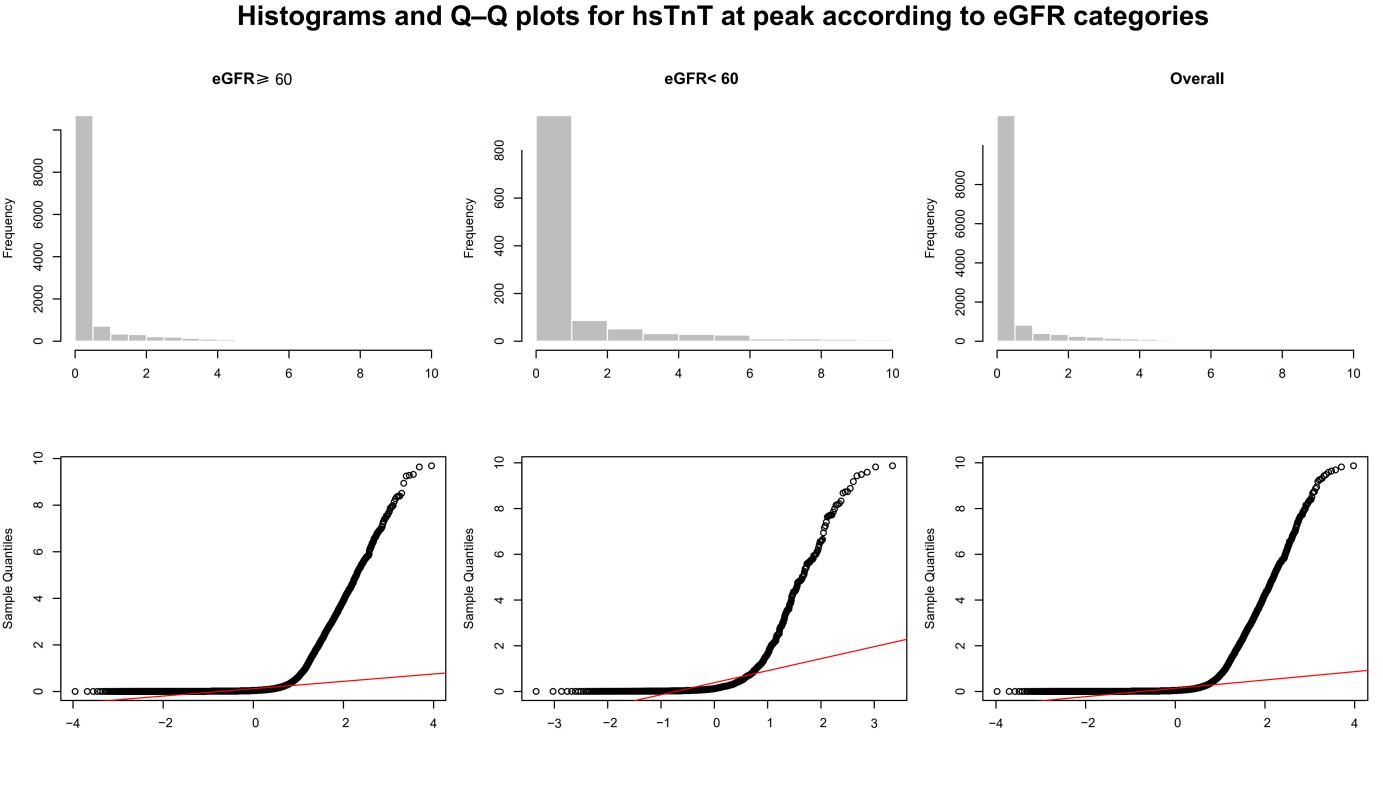


**Figure S5. Histograms and Q-Q plots for number of stents according to eGFR categories.**


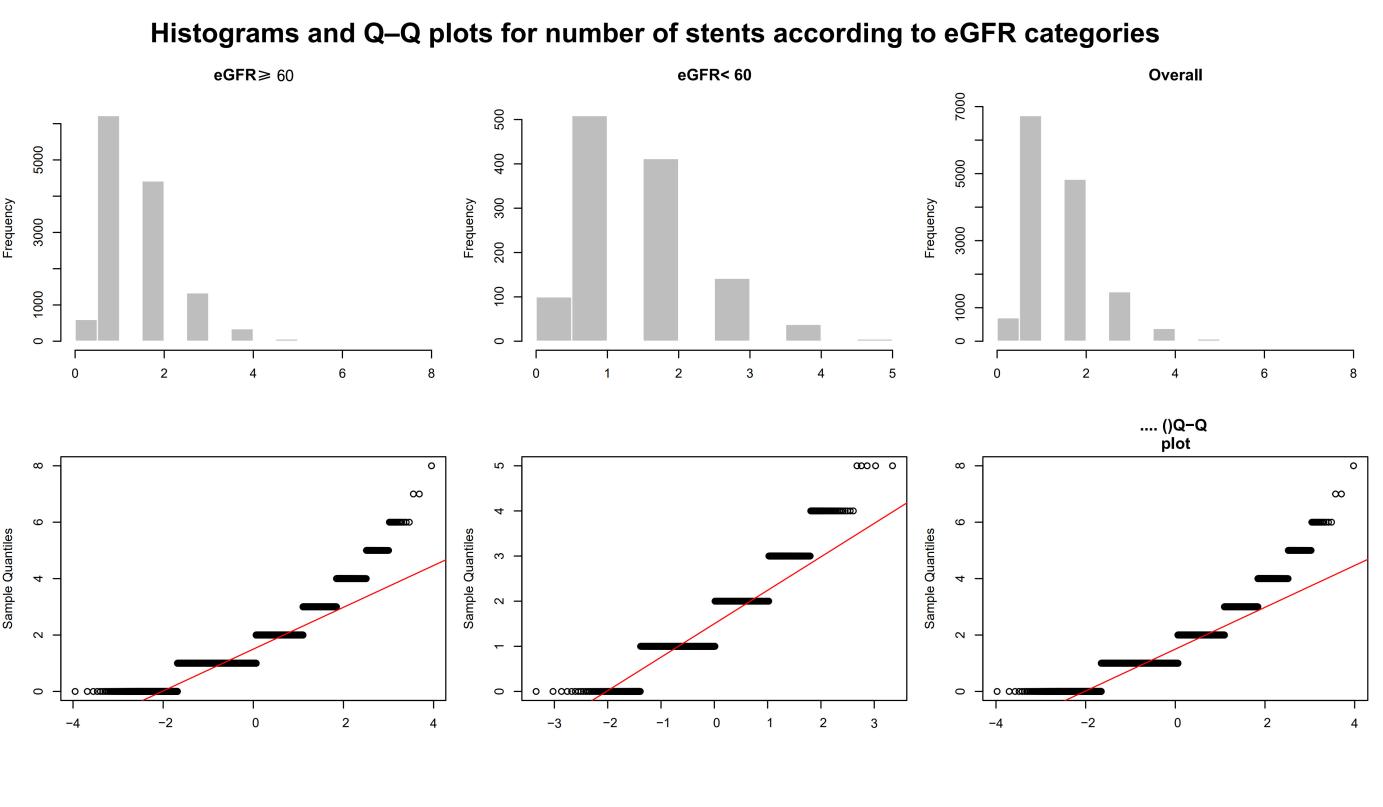


**Figure S6. Histograms and Q-Q plots for total length of stents according to eGFR categories.**


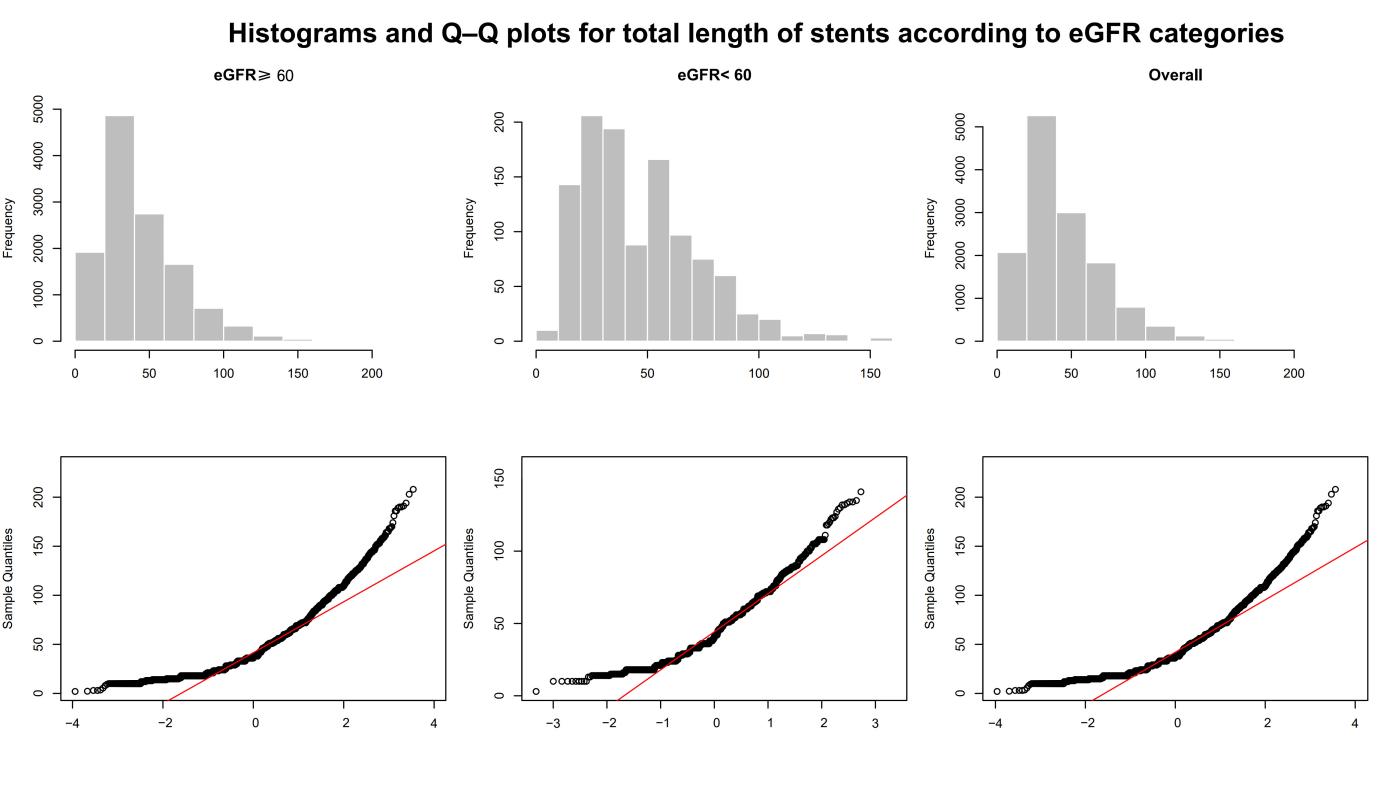


**Figure S7. Histograms and Q-Q plots for average stent diameters according to eGFR categories.**


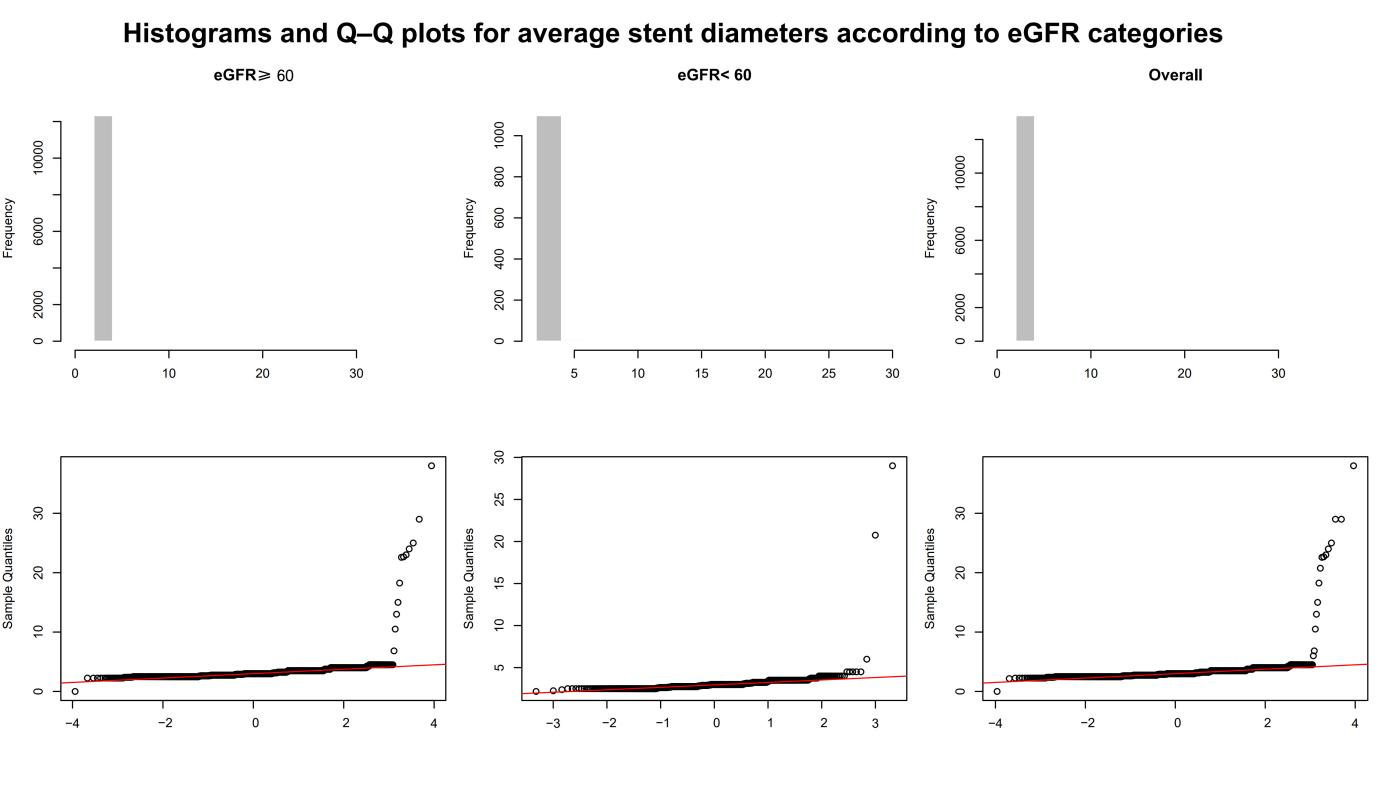

Supplement: Supplementary file 1 [file Datasheet1.docx]
